# Weightlifting during the COVID-19 Pandemic—A Transnational Study Regarding Motivation, Barriers, and Coping of Master Athletes

**DOI:** 10.3390/ijerph18179343

**Published:** 2021-09-04

**Authors:** Marianne Huebner, Wenjuan Ma, Thomas Rieger

**Affiliations:** 1 Department of Statistics and Probability, Michigan State University, East Lansing, MI 48824, USA; 2 Center for Statistical Training and Consulting, Michigan State University, East Lansing, MI 48824, USA; mawenjua@msu.edu; 3 Faculty of Business and Sport, University of Europe for Applied Sciences, 58636 Iserlohn, Germany; thomas.rieger@ue-germany.com

**Keywords:** weightlifting, older athletes, gender differences, pandemic, COVID-19, motivation, barriers, sport

## Abstract

Sport has been heavily impacted by the pandemic for over a year with restrictions and closures of facilities. The main aims of this study are to identify motivation and barriers for an international group of Master weightlifters (ages 35 and up) and analyze age and gender differences in pandemic-related changes to physical activities. A sample of 1051 older athletes, 523 women and 528 men, aged from 35 to 88 years, from Australia, Canada, Europe, and the USA provided responses to an online survey conducted in June 2021. A confirmatory factor analysis was performed to examine age, gender, and regional differences about motivation, barriers, and pandemic impact on sport and physical activities. Participants showed enthusiasm for the opportunity to compete despite health challenges with increasing age but faced barriers due to access to training facilities and qualified coaches even before the pandemic. The oldest athletes had the greatest reduction in physical activities during the pandemic. Weightlifters had the opportunity to compete in virtual competitions and 44% would like to see some of these continued in the future, especially women. These findings highlight the benefits of competitive sports and may provide future directions in strength sports for organizations, sports clubs, and coaches.

## 1. Introduction

Sport has been heavily impacted by the Coronavirus disease. The World Health Organization declared COVID-19 as a pandemic in March 2020 [1]. Associated lockdown restrictions and infection rates differed across regions and resulted in the majority of sport competitions around the world being put on hold. These restrictions extended to closures of training facilities, including private and commercial gyms and weightlifting clubs, as the level of social proximity in those settings is high [2]. Lockdown measures and access to training facilities differed across countries. Due to its island status and rigorous management, including an entry ban, Australia navigated the pandemic, experiencing two waves with higher incidences in March/April and June until August 2020. Facilities in Australia have been allowed to reopen from 8 November 2020 onwards [3]. Thus, weightlifters have been training under relatively normal conditions. The situation in the USA and Canada strongly varies between states and provinces. Many had mandatory closures or were limited to 50% or less capacity in gyms [3]. The lockdown measures were similar in Germany, Spain, and the UK. In Germany, public and commercial sport facilities were fully closed from mid-March until the end of May 2020 and from November 2020 until mid-May 2021. Small clubs are the staple of affordable sport activities in Europe, but for almost 9 months, weightlifters had no access to appropriate training facilities. Starting in June 2021, with decreasing incidence rates, facilities reopened gradually, and sport activities returned. Moreover, competition dates for the second half of 2021 were announced.

Resistance training is a lifelong physical activity commonly reported among the top five physical activities for adults older than 18 years of age in the Americas, Europe, and Western Pacific (5.0–6.6%), and also for adolescents in Eastern Europe (12.4%) [4]. In comparison, team sport activities are low among adults [4]. Weightlifting has enjoyed an unprecedented increase in participation in recent years, especially among women [5]. Weightlifting is an individual sport and it is suitable to train in a number of venues, including at home. However, access to equipment and training with high loads of weight presents a unique problem to this group [2].

Early in the pandemic (April–June 2020), a drop in sport participation and a significant decrease in minutes spent on physical activities per week—especially in older adults (60+ years)—was observed in community dwelling adults in several countries [6,7,8]. USA Weightlifting experienced a one-third drop in membership. Key factors relating to athletes’ motivation to continue training include the availability of competitions [8] and maintaining good health [9]. Weightlifting organizations empowered athletes by offering virtual competitions livestreamed from home gyms or other training locations. The lifts were judged live by three referees, as it would be in an in-person competition. The World Master Weightlifting Championships took place in such a format 24–29 May 2021.

Despite the popularity of resistance training [4], very few strength training athletes were considered in pandemic related studies. This provides a unique opportunity to study Master weightlifters in different countries. Athletes have generally higher levels of self-determination compared to the general population [10,11]. Thus, the main aims of this study are (1) to identify motivation and barriers for Master weightlifters (ages 35 and up) in six countries; (2) to evaluate pandemic-related changes to physical activities after 1 year into the pandemic, examining age and gender differences; and (3) to compare demographics of Master weightlifters in the USA to a pre-pandemic snapshot obtained in January 2020 [12].

## 2. Materials and Methods

### 2.1. Sample

The participants were weightlifters in the Master category who would turn 35 or older during 2021 in Australia (AUS), Canada (CAN), Germany (GER), Great Britain (GBR), Spain (ESP), and the USA. Individuals were invited to participate in the study through email and newsletters from the national governing bodies of weightlifting and via online platforms, including Facebook and Instagram. The survey was administered online via Qualtrics (Provo, UT, USA). The study protocol was approved by the Michigan State University Human Research Ethics Committee and all participants provided online informed consent. Of the 1120 respondents, 65 were excluded from the study due to missing information for pandemic-related questions. Of the 1055 study participants, four indicated their gender as “other” (non-binary, genderqueer, or unspecified), who were included in the descriptive analyses of responses, but excluded from statistical models examining gender effect.

The survey was administered in June 2021 when the pandemic had already been in place for over a year and restrictions had started to ease or were expected to ease. At the time of the survey, the number of cases of COVID-19 infection decreased with the availability of vaccinations, but several areas still experienced lockdown measures. The World Master Weightlifting Championships, which had been cancelled in the previous year, had just finished in a virtual format, and the continental championship had been announced to take place in-person later in the year. During the course of the pandemic, lockdowns and infection rates differed across countries.

### 2.2. Measures

Survey questions on motivation and barriers were developed based on a content analysis of open-ended questions in a survey conducted in the USA pre-pandemic in January 2020, which served as a comparison [12]. Questions related to the impact of the COVID-19 pandemic on exercise were based on participants’ behavior “over the past month.” Questions related to motivation, barriers, and impact of the pandemic had a five-point Likert response scale: 1 = “Strongly agree”, 2 = “Agree”, 3 = “Neither agree nor disagree”, 4 = “Disagree”, and 5 = “Strongly disagree”. The comparison of sport participation during the pandemic to pre-pandemic levels had a five-item response scale: 1 = “A lot more”, 2 = “A little more”, 3 = “About the same”, 4 = “A little less”, and 5 = “A lot less.” The survey questions were presented to the National Master Chairs of each country and were adjusted according to their feedback. The survey was conducted in the official language of each country and was piloted by eight individuals whose responses were not included in the analyses.

### 2.3. Statistical Analysis

Continuous variables were summarized with medians and quartiles and categorical variables were summarized with frequencies and percentages, stratified by gender and age groups. Age groups were defined as 35–44 years, 45–59 years, and 60 years and older. The age groupings were chosen to align with Master age categories in 5-year increments, to separate the younger age group with the largest recent increase in participation, and to study a combined older age group covering a wide age range due to relative paucity of data for older ages. Chi-square tests were used to test differences in categorical variables between women and men. Ordinal logistic regression was used to examine the interest in opportunities to compete as a motivation for Master weightlifting as well as for comparing the volume of sport participation during the pandemic to pre-pandemic levels. Sport participation had a 5-item response scale from “a lot more” to “a lot less.” Age, gender, and geographic region were the independent variables in the models.

Likert scale measure items were used to model constructs. We used confirmatory factor analysis (CFA) [13] to form six constructs, including motivations for Master weightlifting, socio-economic barriers, health-related barriers, training access barriers, pandemic impact on exercise routines, and health reasons for training during the pandemic. Group CFA models [14,15,16,17] were used to compare the differences of the constructs across gender, age groups (35–44, 45–59, 60+), and geographic regions (Australia, Europe, Canada, and the USA). We first established measurement invariance by fixing a series of parameters equal across groups, including factor loadings, thresholds, and factor covariances. We then compared the levels of the constructs across the groups. Model fit was evaluated with the chi-square ratio and degrees of freedom (χ^2^/*df*), the comparative fit index (CFI), and the approximation mean square error (RMSEA).

Statistical analyses were performed utilizing the statistical software R v. 4.0.3 [18]. Mplus 8.6 [19] was used for the CFA models. For all analyses, a *p*-value of 0.05 was considered statistically significant. The study was reported according to the STROBE statement [20].

## 3. Results

**Figure 1:**
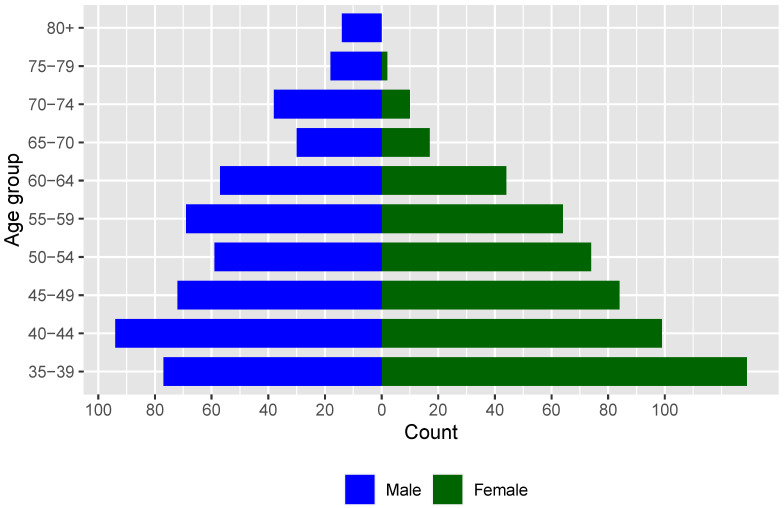
Age pyramid of survey participants.

Table 1 lists demographics by region and gender of the 1055 participants from Australia (8.6%), Canada (11.8%), Europe (17.7%), and the USA (61.9%). Overall, 523 women participated in the survey (49.7%). There were more than 50% women in all regions, except in Europe, where women accounted for only 31% of the participants. This was the case in Germany and in Spain, but not in the UK. The mean age of men was higher than of women in all regions. While female participation in weightlifting surpassed that of male in the younger age groups (from 35 to 44 years old) in all regions, female participation in weightlifting was still sparse in the oldest age groups (Figure 1). Master weightlifters were highly educated. The training location varied across regions, but more women than men trained in CrossFit boxes. A large majority of women worked with in-person coaches, and men were more likely than women to have their own program. Overall, 25.3% of the participants made use of remote coaching.

### 3.1. Attitudes toward Sport and Physical Activity during the Pandemic

**Figure 2:**
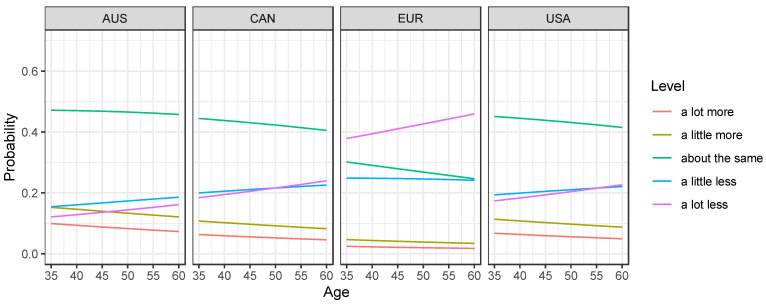
Estimated probability of sport and physical activities during the pandemic compared to a typical week prior to the pandemic.

There were regional differences in the responses to the question *“Did the priority you place on physical activities change during the pandemic?”* (*p* = 0.015). It was more likely to increase in Canada (23.8%) and Australia (25.6%), while 21.7% of European participants reported that their priority decreased. The participation in sport or physical activities also differed by region, corresponding to the question *“During the pandemic, did you participate in sport or physical activities more, less, or about the same compared to a typical week prior to the pandemic?”* (*p* < 0.001). Compared to pre-pandemic levels, “a lot less” sport activity was reported by participants in Australia (14.3%), Canada (18.7%), the USA (20.4%), and Europe (44.4%). Older athletes were more likely to report a lot less sport activity with an odds ratio (OR) for age in 5-year increments of 1.07 (95% CI: 1.02, 1.12, *p* = 0.009) (Figure 2). This was particularly the case for older athletes in Europe, where 58.3% reported a lot less sport activity for participants aged 60 or older, while it was 33.9% for 35–44 year-olds and 46.0% for 45–59 year-olds. Post-pandemic, 50% of participants in Europe want to increase their sport and exercise regime, 43% in Canada, 34% in the USA, and 19% in Australia (regional differences: *p* < 0.001).

### 3.2. Motivation, Barriers, and Pandemic Impact on Exercise

Participants selected “agree” or “strongly agree” for several factors that attract them to Master weightlifting (Table 2). These factors included maintaining fitness (95.1%), building strength and confidence (93.4%), and the opportunity to compete (90.3%), which were similar across age groups. Several barriers exist, notably career and work responsibilities (53.8%) and family responsibilities (36.9%). This was more common in age groups below 60 years. Older athletes were more affected by injuries (51.3%) and health issues (20.7%) compared to younger athletes.

Four CFA models were developed with six constructs each—an overall model (Table 3) and group models comparing women versus men; age groups 35–44, 45–59, and 60 and older; and geographic regions (Table 4). Model fit indices are reported in Table A1 and correlations between constructs are in Table A2.

In the group CFA models, the means of the constructs of the reference group are fixed as 0. Therefore, the tests of the means of comparison groups are the statistical tests of the differences between the comparison group and the reference group (Table 4). Since the response scale was from 1 = “strongly agree” to 5 = “strongly disagree”, lower numbers indicate a stronger endorsement of the statement.

Health reasons such as injuries were noted as a barrier to weightlifting at older ages compared to the 35–44 age group. Exercising to manage physical and mental health during the pandemic was less often selected by weightlifters aged 60 or older compared to younger weightlifters. Also, socio-economic factors were less of a concern for this age group.

There were gender differences for motivation and health concerns. Women more commonly identified social aspects, building strength and confidence, managing stress, and staying fit as attractions to the sport of Master weightlifting compared to men. During the pandemic, women were more concerned with exercising due to managing physical and mental health reasons than men. Men noted injuries or other health reasons as barriers to Master weightlifting more frequently than women.

There were also regional differences for the pandemic impact on exercise; European weightlifters were especially negatively impacted, while Australians were more likely to continue with their exercise regime compared to USA weightlifters. However, Europeans were less likely to indicate barriers to Master weightlifting due to finances, work, or family-related responsibilities. Canadian Master weightlifters were similar in all aspects to their USA counterparts.

### 3.3. Opportunities for Competitions

**Figure 3:**
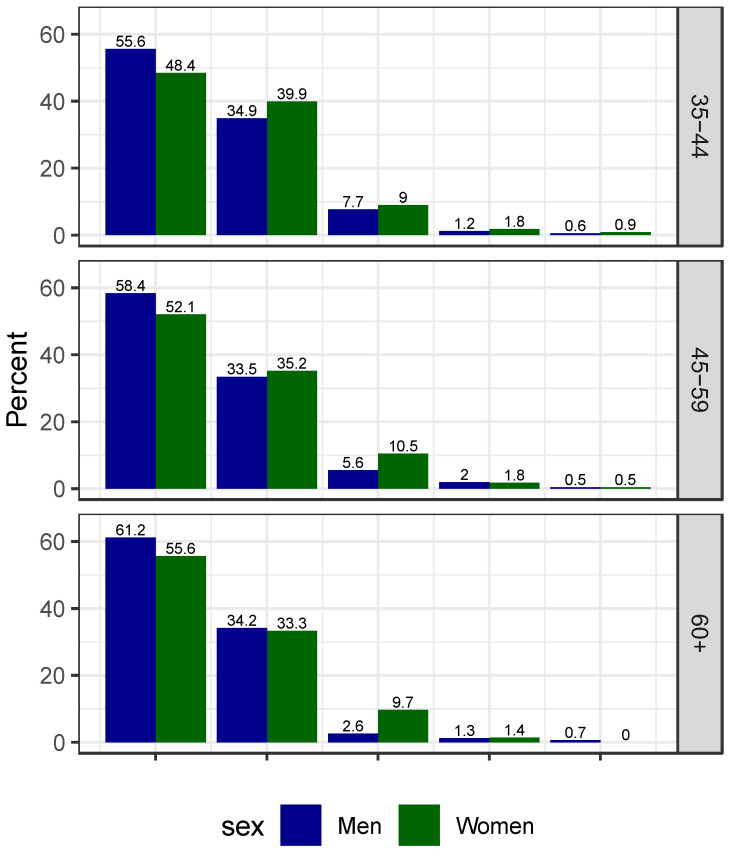
Percentages of participants attracted to Master weightlifting because of the opportunity to compete stratified by gender and age group. The responses were “strongly agree”, “agree”, “Neither agree nor disagree”, “disagree”, “strongly disagree” for the bars from left to right.

Master weightlifters are attracted to the sport by the opportunity to compete (90.2% agree or strongly agree), and this was similar across age groups (Figure 3). Men were more motivated than women (*p* = 0.022). Australian participants were less motivated by competition opportunities than USA participants (*p* = 0.0017), while there were no significant differences between the other regions (Table A3).

**Figure 4:**
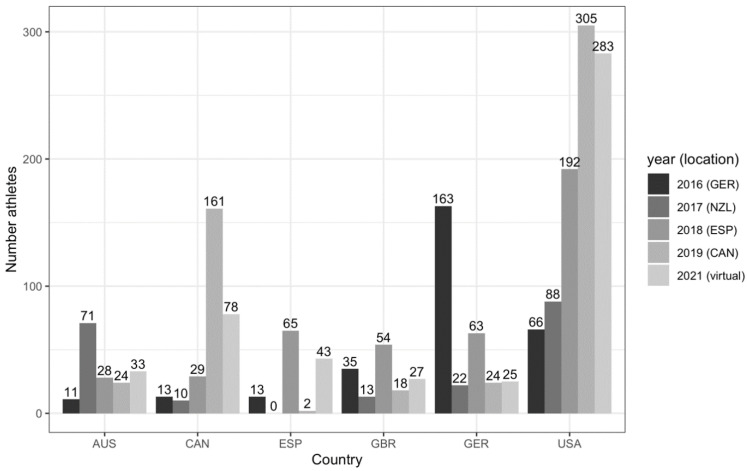
Participation in the World Master Weightlifting Championships from 2016 to 2021 for the six countries in this survey.

The World Master Weightlifting Championships in 2021, took place in a virtual format 24–29 May 2021, with 763 competitors from 60 countries. These championships included athletes from more countries than in previous years. Participation is usually more accessible to athletes in the host countries (Figure 4). There were no World Master Championships in 2020 due to the pandemic, but there were 719 competitors from 50 countries in Canada in 2019 and there were 777 competitors from 48 countries in Spain in 2018.

Standardized performances can be compared between the in-person and virtual world championships in 2019 and 2021, respectively. This is done by taking body weight into account and adjusting for age [5,21] to calculate the total weight (in kg) that athletes would be able to lift if they had the same body weight and age. There was no difference in standardized performance from 2019 to 2021. The median (quartiles) of the standardized performances for women (‘SHMF’) was 222.9 (200.2, 245.6) in 2019 and 224.0 (202.4, 245.7) in 2021 (*p* = 0.767). For men (‘SMF’), it was 323.2 (301.7, 346.5) in 2019 and 320.8 (298.6, 345.1) in 2021 (*p* = 0.286).

The question *‘Which weightlifting-specific events would you like to see continue after the pandemic?’* with options ‘virtual qualifying competitions,’ ‘virtual championships,’ ‘webinars,’ and ‘other’ was answered by 827 of the 1055 participants. Of all the participants, 43.7% would like to have future virtual competitions. More women than men chose this option (60.3% vs. 39.7%). Canadian respondents were especially in favor (69.4%), while only 23.8% of the German respondents chose this option. In contrast, only 21.0% would like to continue with virtual championships; concerns regarding cheating and doping were mentioned in the comments. A strong preference for not continuing with any form of virtual competitions was expressed by 173 respondents in comments (16.4%). Continuing education via webinars found favor with 39.7%. This was especially the case for German respondents (52.3%). Very few respondents mentioned specific topics; among these were coaching courses, education of technical officials, Master training themes, and seminars for women.

### 3.4. Comparison to Pre-Pandemic in the USA

A survey of USA Master weightlifters was conducted in January 2020, which can serve as a reference for the pre-pandemic situation [12]. Various states in the USA approached COVID lockdown measures differently, starting in March 2020. By June 2021, most states had loosened COVID restrictions either partially or completely. There were 652 survey participants in June 2021 compared to 951 in January 2020. This is a 31% drop, but it is consistent with about a one-third drop in membership to USA Weightlifting (USAW) during the pandemic. Demographic differences are described in Table A4.

After over 1 year into the pandemic, the age of survey participants shifted to the older ages; there were 39.4% in the 35–44 age group compared to 47.3% pre-pandemic, while there were 21.4% in the 60+ age group compared to 14.3% pre-pandemic. Participants in the June 2021 survey had more years of experience in weightlifting (median of 7 years of experience) than participants in the pre-pandemic survey (median of 4 years of experience). More participants followed their own training program during the pandemic (26.2%) or in combination with other programs than before (17.8%). About the same proportion trained at home (31% vs. 30%).

## 4. Discussion

The key objectives of this study were to identify motivation and barriers for Master weightlifters (ages 35 and up) in six different countries and to explore responses to pandemic-related changes in physical activities after 1 year into the pandemic. The findings indicate that older athletes have a high level of self-determination and are attracted to the sport for health and fitness reasons, to build strength and confidence, and because of the opportunity to compete, while health challenges with increasing age and access to appropriate training facilities and qualified coaches knowledgeable about the needs of older athletes constitute barriers. The pandemic affected weightlifters in different countries unequally, with a larger impact on sport and physical activities in Europe. The largest reduction in physical activities was in the oldest age groups, but more years of experience in weightlifting may have been favorable to stay active in the sport after over 1 year into the pandemic. Women were more likely to be motivated to exercise to manage physical and mental health during the pandemic. Given the dearth of literature in Master sports within the context of the global pandemic, this is a timely study to understand implications for future actions and policies of organizations, sport clubs, coaches, and athletes.

Sport is an area heavily impacted by the pandemic due to closures or restrictions of training facilities and cancellations or postponement of sport events [22]. Detraining effects began after a few weeks [2], with a larger effect in older athletes [23]. Several studies on pandemic impact on athletes in training for various sport disciplines was done at the beginning of the pandemic when it was unpredictable how long it might last [8,9,10]. The athletes in these studies were usually younger than participants in our study. Other studies, not specific to athletes, conducted online surveys in broader community-dwelling older adults also at the beginning of the pandemic [6,7,24]. There is a lack of studies that evaluate motivation and commitment, which are especially important during the pandemic to identify population subgroups for targeted interventions [9]. At the time of our study on older athletes in weightlifting, the pandemic had been in place for over a year and restrictive measures had started to ease or were expected to ease. This study contributes to the knowledge of how older athletes have coped for an extended period during the pandemic and provides a baseline to study possible longer-term effects.

Several factors make Master weightlifting attractive to older athletes. Maintaining fitness, building strength and confidence, and the opportunity to compete were selected by more than 90% of the participants, closely followed by participating in a community and managing stress with 84% and 73%, respectively. This was especially the case for women who accounted for most participants in the youngest age group (35–44 years), although they were less represented at age 60 and older. This is congruent with other studies which noted that several factors stimulate physical activity, such as physical (fitness, strength), psychological (reduce stress, self-esteem), and socio-ecological (socializing) factors [9]. Furthermore, a key factor in athletes’ motivation to continue training is the availability of competitions, and thus these provide a unique benefit to athletes [8,25]. In our study, health challenges constituted a barrier to sport participation with increasing age. Lack of access to training facilities and qualified coaches was selected by more than 20% of the participants, and this was similar for all age groups, geographic regions, and gender for these older athletes. Women were more likely to select managing physical and mental health as a reason to exercise during the pandemic. Physical and psychological benefits and overcoming barriers due to health were aspects identified in a study on Master athletes participating in World Master Games [26,27]. Physical and mental health were also valued by younger participants in a university population with a high commitment to physical activity [9].

The pandemic impact on exercise has been substantial due to closures of training facilities. This was especially the case for European weightlifters who experienced a larger impact on exercise routines and trained a lot less compared to pre-pandemic levels. Coping strategies for weightlifters during the pandemic were the use of online tools (37%), remote coaching (25%), and finding new ways to be physically active (55%). A study of a younger population of university students (amateur and recreation athletes) reported several coping strategies, mainly using online tools for exercise and for keeping socially connected [10]. More than half of the weightlifters in this study missed the physical activities they used to do, but also found new ways to engage in PA. Following gym closures and distancing requirements, it may have become necessary to train alone, but 32% of the weightlifters indicated that they do not enjoy training alone. However, such a change also confers advantages with respect to choosing training times. This was noted in an Australian study where athletes indicated more flexibility in training times shifting towards morning and afternoon sessions instead of evenings and the duration of training sessions decreased during lockdown restrictions [8]. In a Danish population, older adults experienced a significant decrease in leisure physical activity, and the highest drop was seen among those with lower levels of education [6]. Although Master athletes in general have high levels of education [12,28], including in this study, older weightlifters reported less participation in sport and physical activities compared to pre-pandemic levels than younger age groups. Interestingly, comparing the demographics to a pre-pandemic survey in the USA in January 2020, the proportion of 60 years and older participants was higher in the present survey conducted in June 2021. Participants had more years of experience in weightlifting than participants in the pre-pandemic survey. This may indicate a high commitment level or self-determination, which are favorable for the continuation of training [10,11].

While the pandemic impacted weightlifters unequally, the virtual competitions motivated them to keep training at high levels and opened avenues for participation to some who would not have been able to compete otherwise due to the time and travel costs involved, as can be seen from athletes representing more countries, and their performances were comparable to pre-pandemic levels in the virtual World Master Weightlifting Championships. A large proportion of participants in this survey would like to see the continuation of such virtual events. The performances in the world championships in 2019 and the virtual one in 2021 were comparable on average, indicating that the athletes were able to train at a high level. This may have been possible due to more athletes having access to strength equipment at home that can help maintain minimum performance levels [2]. Programs to support grassroots efforts and coaches would likely improve engagement. Sport organizations play a central role in creating opportunities for online competitions or continuing education, although these are not substitutes for in-person events where social interactions are valued.

Strengths and limitations. The data collection took place after more than 1 year since the start of the pandemic. There was attrition of master athletes in national organizations and there could be a selection bias of who stayed or started up again compared to surveys prior to the pandemic. However, we were able to compare the demographics to baseline data from a USA survey conducted just prior to the pandemic in January 2020. The methods of dissemination of the survey via email and social media differed between countries but reflected typical formats of communication between the national organizations and their members. All data are self-reported, and this can lead to bias. Since the sport of weightlifting has seen an unprecedented increase in participation rate in recent years, especially for women, this study provides a much-needed baseline to study the long-term impact of the pandemic. We believe that by including several countries, we have increased the external validity of the findings. Countries with large participation rates of both genders in World Master Weightlifting Championships were chosen for this survey. Future research should include all countries of the IWF Masters.

## 5. Conclusions

One year into the pandemic, Master weightlifters are finding new ways to train; training in different locations, including at home; using online tools; and some are able to participate in virtual competitions. Strong motivators for staying active in the sport are being able to compete, building strength and maintaining fitness, managing stress, and social aspects. Barriers are health, family and work responsibilities, and access to training facilities. The long-term impact of the pandemic on sport participation should be a topic of future research. This is especially the case for the oldest group of athletes who have experienced the largest reduction in physical activities.

## Figures and Tables

**Table 1:** Demographics of Master Weightlifters across Australia, Canada, the USA, and Europe.

	*N*	AUS (*n* = 90)		CAN (*n* = 124)		USA (*n* = 652)		EUR (*n* = 189)	
	1055 ^1^	Women (*n* = 46)	Men (*n* = 44)	Women (*n* = 69)	Men (*n* = 55)	Women (*n* = 350)	Men (*n* = 302)	Women (*n* = 59)	Men (*n* = 130)
Age ^2^	1055	39 47 54	42 48 62	40 45 53	45 54 62	40 46 55	41 49 62	40 45 54	44 53 60
Age group	1055								
35–44	401	43.5% (20)	36.4% (16)	47.8% (33)	21.8% (12)	42.6% (149)	35.7% (107)	44.8% (26)	27.9% (36)
45–59	424	39.1% (18)	31.8% (14)	37.7% (26)	40.7% (22)	43.1% (151)	34.7% (104)	46.6% (27)	46.5% (60)
60+	230	17.4% (8)	31.8% (14)	14.5% (10)	38.2% (21)	14.3% (50)	29.7% (89)	8.6% (5)	25.6% (33)
Age at start of weightlifting ^2^	1043	35 40 48	30 37 48	34 40 45	15 33 45	34 39 48	21 34 43	31 38 42	13 16 30
Education	1051								
Low	91	10.9% (5)	6.8% (3)	5.8% (4)	18.5% (10)	5.5% (19)	6.7% (20)	13.8% (8)	17.2% (22)
Middle	187	26.1% (12)	22.7% (10)	34.8% (24)	27.8% (15)	9.2% (32)	9.7% (29)	24.1% (14)	39.1% (50)
High	390	58.7% (27)	61.4% (27)	50.7% (35)	46.3% (25)	37.9% (132)	33.3% (100)	32.8% (19)	18.8% (25)
Graduate degree	383	4.3% (2)	9.1% (4)	8.7% (6)	7.4% (4)	47.4% (165)	50.3% (151)	29.3% (17)	25.0% (32)
Training location ^3^	1046								
Weightlifting club	605	58.7% (27)	45.5% (20)	66.7% (46)	71.7% (38)	53.0% (185)	48.1% (143)	75.9% (44)	80.2% (101)
CrossFit box	452	45.7% (21)	36.4% (16)	47.8% (33)	24.5% (13)	53.3% (186)	45.5% (135)	43.1% (25)	17.5% (22)
Fitness center	178	15.2% (7)	22.7% (10)	17.4% (12)	15.1% (8)	15.2% (53)	21.9% (65)	15.5% (9)	10.3% (13)
Home	305	34.8% (16)	45.5% (20)	17.4% (12)	47.2% (25)	22.1% (77)	41.8% (124)	10.3% (6)	17.5% (22)
Training program ^4^	1049								
Coach	600	87.0% (40)	50.0% (22)	71.0% (49)	48.1% (26)	68.6% (240)	44.5% (133)	63.8% (37)	38.9% (49)
Remote coach	196	15.2% (7)	13.6% (6)	15.9% (11)	18.5% (10)	23.5% (82)	21.7% (65)	15.5% (9)	4.0% (5)
Own	331	4.3% (2)	38.6% (17)	13.0% (9)	46.3% (25)	10.0% (35)	45.2% (135)	31.0% (18)	71.4% (90)

Abbreviations: PA = physical activity. ^1^ participants included 4 with gender as “other”; ^2^ *a b c =* the lower quartile *a*, the median *b*, and the upper quartile *c* for continuous variables; % may add to more than 100%, since a combination of locations or programs possible.^3, 4^.

**Table 2:** Master weightlifting attractions and barriers (“strongly agree” or “agree”).

	*N*	35–44 (*n* = 401)	45–59 (*n* = 424)	60+ (*n* = 230)	Total (*n*= 1055)
**Motivations**					
Community/social aspects	1043	72.4% (288)	74.2% (313)	73.5% (164)	73.3% (765)
Opportunity to compete	1036	89.3% (352)	89.5% (374)	93.3% (209)	90.3% (935)
Manage stress/mental health	1034	88.2% (350)	86.4% (363)	71.4% (155)	83.9% (868)
Build strength, confidence, positive identity	1037	94.2% (374)	94.0% (394)	91.0% (201)	93.4% (969)
Health/Fitness	1039	93.7% (371)	95.7% (401)	96.4% (216)	95.1% (988)
**Barriers**					
Finances	1037	24.9% (99)	21.8% (91)	18.6% (41)	22.3% (231)
Family responsibilities	1035	45.1% (179)	38.4% (160)	19.5% (43)	36.9% (382)
Career/work responsibilities	1038	61.8% (246)	55.3% (231)	36.5% (81)	53.8% (558)
Access to a gym	1030	19.0% (75)	22.8% (95)	16.5% (36)	20.0% (206)
Access to qualified coaches	1031	24.1% (95)	27.8% (116)	20.2% (44)	24.7% (255)
Injuries/surgery	1039	46.9% (187)	51.7% (217)	58.6% (129)	51.3% (533)
Other health issues	1020	14.4% (57)	23.4% (97)	27.3% (57)	20.7% (211)

**Table 3:** Description of the CFA constructs and results.

	Loading	SE	*p*-Value	STD Loading
**1. Motivation for weightlifting**				
Community/social aspects	1.000	0.000	---	0.410
Manage stress/mental health	1.886	0.150	0.000	0.774
Build strength, confidence, positive identity	2.162	0.170	0.000	0.888
Health/fitness	1.842	0.146	0.000	0.756
**2. Socio-economic barriers**				
Finances	1.000	0.000	---	0.639
Family responsibilities	1.262	0.054	0.000	0.806
Career/work responsibilities	1.168	0.054	0.000	0.746
**3. Health-related barriers**				
Injuries/surgery	1.000	0.000	---	0.619
Other health issues	1.427	0.132	0.000	0.883
**4. Training access barriers**				
Access to a gym	1.000	0.000	---	0.898
Access to qualified coaches	0.788	0.043	0.000	0.708
**5. Pandemic impact on exercise routine**				
The pandemic has not changed my exercise regime	1.000	0.000	---	0.703
During the pandemic, I found new ways to be physically active	0.472	0.051	0.000	0.331
I have missed doing the types of physical activities I did before (R)	0.960	0.064	0.000	0.674
I do not enjoy training by myself (R)	0.459	0.052	0.000	0.323
During the pandemic, I have had more time for sport	0.702	0.054	0.000	0.493
**6. Exercise and motivation during the pandemic**				
I exercised to manage my physical health during the pandemic	1.000	0.000	---	0.920
I exercised to manage my mental health during the pandemic	1.004	0.034	0.000	0.924

**Table 4:** Differences between group means by gender, age, and region for each of the six constructs.

	Motivation for Weightlifting	Barriers to Weightlifting			Pandemic Impact	
		Socio-Economic	Training Access	Health-Related	Exercise Routine	Exercise and Motivation
**Gender comparison: (men as reference group)**						
**Women**						
Estimate	**−0.229**	−0.081	−0.068	**0.112**	0.067	**−0.430**
SE	0.040	0.044	0.065	0.046	0.055	0.071
*p*-value	<0.001	0.066	0.295	0.014	0.230	<0.001
**Age comparison: (age 35–44 as reference group)**						
**Age 45–59**						
Estimate	−0.005	0.079	−0.112	**−0.124**	−0.010	0.078
SE	0.035	0.051	0.072	0.051	0.059	0.077
*p*-value	0.895	0.119	0.119	0.014	0.868	0.310
		**Age 60+**				
Estimate	0.106	**0.362**	0.059	**−0.208**	−0.010	**0.310**
SE	0.041	0.062	0.088	0.063	0.070	0.089
*p*-value	0.100	<0.001	0.503	0.001	0.888	<0.001
**Region comparison: (USA as reference group)**						
**Canada**						
Estimate	−0.029	0.043	−0.093	0.026	0.112	−0.124
SE	0.060	0.071	0.103	0.065	0.078	0.103
*p*-value	0.627	0.538	0.367	0.691	0.149	0.227
**Europe**						
Estimate	0.060	**0.271**	−0.076	0.018	**0.624**	**0.482**
SE	0.046	0.067	0.081	0.067	0.084	0.095
*p*-value	0.188	<0.001	0.350	0.794	<0.001	<0.001
**Australia**						
Estimate	**0.124**	0.075	−0.128	−0.073	**−0.196**	0.016
SE	0.060	0.076	0.09	0.076	0.085	0.139
*p*-value	0.039	0.324	0.153	0.334	0.021	0.909

## Data Availability

Data supporting the reported results are available on the Dryad platform: https://doi.org/10.5061/dryad.qfttdz0ht, accessed on 7 August 2021.

## Appendix A

The following are the results from the confirmatory factor analysis about motivation, barriers, and pandemic impact. We ran four CFA models with six constructs: overall and group models by gender, age, and geographic region.

**Table A1:** Description of model fit indices of CFA models.

Model	Overall Model	Group Model by Gender	Group Model by Age Group	Group Model by Region
Chi-square	1109.16	1121.230	1266.784	1492.392
Degree of Freedom	120	321	520	687
*p*-value	0.000	0.000	0.000	0.000
Chi-square contributed by female	---	623.829	---	---
Chi-square contributed by male	---	497.401	---	---
Chi-square contributed by 35–44 years of age	---	---	442.294	---
Chi-square contributed by 45–59 years of age	---	---	437.974	---
Chi-square contributed by 60+ years of age	---	---	386.516	---
Chi-square contributed by USA	---	---	---	515.768
Chi-square contributed by Canada	---	---	---	269.026
Chi-square contributed by Europe	---	---	---	399.512
Chi-square contributed by Australia	---	---	---	308.086
RMSEA	0.088	0.069	0.064	0.067
CFI	0.921	0.935	0.942	0.920

**Table A2:** Covariance and correlations between factors and residual variance.

Covariance/Correlation	Covariance	SE	*p*-Value	Correlation
**Pandemic impact on exercise routines WITH**				
Motivation for Master weightlifting	0.006	0.012	0.616	0.021
**Health reasons for exercise during the pandemic WITH**				
Motivation for Master weightlifting	0.181	0.019	<0.001	0.479
Pandemic weight-lifting routine	0.314	0.025	<0.001	0.486
**Socio-economic barriers WITH**				
Motivation for Master weightlifting	0.010	0.011	0.357	0.038
Pandemic impact on exercise routines	−0.062	0.018	0.001	−0.138
Health reasons for exercise during the pandemic	−0.020	0.022	0.357	−0.034
**Training access barriers WITH**				
Motivation for Master weightlifting	0.010	0.011	0.144	−0.060
Pandemic impact on exercise routines	−0.169	0.025	<0.001	−0.268
Health reasons for exercise during the pandemic	0.013	0.031	0.665	0.016
Socio-economic barriers	0.301	0.021	<0.001	0.524
**Health-related barriers WITH**				
Motivation for Master weightlifting	−0.013	0.011	0.232	−0.052
Pandemic impact on exercise routines	−0.052	0.018	0.004	−0.120
Health reasons for exercise during the pandemic	−0.039	0.022	0.075	−0.068
Socio-economic barriers	0.144	0.020	<0.001	0.364
Training access barriers	0.243	0.027	<0.001	0.437
**Residual variance**				
Motivation for Master weightlifting	0.169	0.026	<0.001	---
Pandemic impact on exercise routines	0.494	0.040	<0.001	---
Health reasons for exercise during the pandemic	0.847	0.030	<0.001	---
Socio-economic barriers	0.408	0.030	<0.001	---
Training access barriers	0.807	0.044	<0.001	---
Health-related barriers	0.383	0.041	<0.001	---

Ordinal logistic regression models were used to examine differences between ages, gender, and regions for sport participation and motivation for participation in Master weightlifting because of the opportunity to compete. Results are reported in Table A3.

**Table A3:** Odds ratios (OR) and 95% confidence intervals to evaluate differences in sport participation and motivation to compete.

	Participation in Sport and Physical Activities during the Pandemic (*n* = 1037)	Attracted to Master Weightlifting Due to Opportunity to Compete (*n* = 1036)
*Response scale*	*From 1 = a lot more to 5 =a lot less*	*From 1 = strongly agree to 5 = strongly disagree*
Age, 5 years	1.07 (1.02, 1.12) *p* = 0.008	0.95 (0.90, 1.01) *p* = 0.078
Gender, male	---	0.75 (0.59, 0.96) *p* = 0.024
Region		
USA	ref	ref
CAN	1.07 (0.76, 1.52) *p* = 0.683	0.99 (0.67, 1.45) *p* = 0.946
EUR	2.90 (2.12, 3.95) *p* < 0.001	1.02 (0.76, 1.47) *p* = 0.721
AUS	0.66 (0.44, 0.97) *p* = 0.037	1.74 (1.14, 2.63) *p* = 0.009

A USA Master weightlifting survey was conducted in January 2020. The demographics compared to the 2021 survey demographics are listed in Table A4.

**Table A4:** Comparison of demographics to pre-pandemic Master weightlifters in the USA.

	January 2020 (*n* = 951)	June 2021 (*n* = 652)
Gender		
Women	54.7% (519)	53.7% (350)
Men	45.3% (429)	46.3% (302)
Age	39 45 54	40 48 58
Age group		
35–44	47.3% (450)	39.4% (256)
45–59	38.4% (365)	39.2% (255)
60+	14.3% (136)	21.4% (139)
Education		
Low	3.2% (30)	6.0% (39)
Middle	14.7% (140)	9.5% (62)
High	39.6% (377)	35.7% (232)
Graduate degree	42.5% (404)	48.8% (317)
Experience, years ^1^	1 4 6	4 7 11
Training location		
WL club	47.0% (447)	50.3% (328)
CrossFit box	41.6% (396)	49.5% (323)
Fitness center	13.4% (127)	18.1% (118)
Home	29.9% (284)	30.8% (201)
Training program		
Coach	75.8% (721)	75.5% (492)
Own program	17.8% (169)	26.2% (171)

*^1^ a**b**c =* the lower quartile *a*, the median *b*, and the upper quartile *c* for continuous variables.
